# Multidimensional Diffusion Magnetic Resonance Imaging for Characterization of Tissue Microstructure in Breast Cancer Patients: A Prospective Pilot Study

**DOI:** 10.3390/cancers13071606

**Published:** 2021-03-31

**Authors:** Isaac Daimiel Naranjo, Alexis Reymbaut, Patrik Brynolfsson, Roberto Lo Gullo, Karin Bryskhe, Daniel Topgaard, Dilip D. Giri, Jeffrey S. Reiner, Sunitha B. Thakur, Katja Pinker-Domenig

**Affiliations:** 1 Memorial Sloan Kettering Cancer Center, Department of Radiology, Breast Imaging Service, 300 E 66th Street, New York, NY 10065, USA; isaac.daimiel@gmail.com (I.D.N.); logullor@mskcc.org (R.L.G.); reinerj@mskc.org (J.S.R.); thakurs@mskcc.org (S.B.T.); 2 Department of Radiology, Breast Imaging Service, Guy’s and St. Thomas’ NHS Trust, Great Maze Pond, London SE1 9RT, UK; 3 Random Walk Imaging AB, SE-22002 Lund, Sweden; alexis@rwi.se (A.R.); patrik@rwi.se (P.B.); karin@rwi.se (K.B.); 4 NONPI Medical AB, SE-90738 Umeå, Sweden; 5 Department of Chemistry, Lund University, SE-22100 Lund, Sweden; daniel.topgaard@fkem1.lu.se; 6 Memorial Sloan Kettering Cancer Center, Department of Pathology, 1275 York Ave, New York, NY 10065, USA; girid@mskcc.org; 7 Memorial Sloan Kettering Cancer Center, Department of Medical Physics, 1275 York Ave, New York, NY 10065, USA

**Keywords:** diffusion-weighted imaging, magnetic resonance imaging, breast cancer, multidimensional diffusion MRI, oscillating gradients

## Abstract

**Simple Summary:**

In this prospective pilot study, we investigated the potential of clinical multidimensional diffusion magnetic resonance imaging (MDD MRI) for the microstructural characterization of breast cancers and normal fibroglandular breast tissue. The method relies on advanced gradient waveforms to encode the signal with information about cell densities, shapes, and orientations, and to quantify tissue composition as a probability distribution of diffusion tensors in a space with dimensions analogous to those on a cellular level. Sixteen patients with breast cancer underwent MDD MRI within a clinically feasible scan time of approximately 4 min, providing voxel-resolved distributions and parameter maps with microstructural information that is not accessible with conventional methods.

**Abstract:**

Diffusion-weighted imaging is a non-invasive functional imaging modality for breast tumor characterization through apparent diffusion coefficients. Yet, it has so far been unable to intuitively inform on tissue microstructure. In this IRB-approved prospective study, we applied novel multidimensional diffusion (MDD) encoding across 16 patients with suspected breast cancer to evaluate its potential for tissue characterization in the clinical setting. Data acquired via custom MDD sequences was processed using an algorithm estimating non-parametric diffusion tensor distributions. The statistical descriptors of these distributions allow us to quantify tissue composition in terms of metrics informing on cell densities, shapes, and orientations. Additionally, signal fractions from specific cell types, such as elongated cells (bin1), isotropic cells (bin2), and free water (bin3), were teased apart. Histogram analysis in cancers and healthy breast tissue showed that cancers exhibited lower mean values of “size” (1.43 ± 0.54 × 10^−3^ mm^2^/s) and higher mean values of “shape” (0.47 ± 0.15) corresponding to bin1, while FGT (fibroglandular breast tissue) presented higher mean values of “size” (2.33 ± 0.22 × 10^−3^ mm^2^/s) and lower mean values of “shape” (0.27 ± 0.11) corresponding to bin3 (*p* < 0.001). Invasive carcinomas showed significant differences in mean signal fractions from bin1 (0.64 ± 0.13 vs. 0.4 ± 0.25) and bin3 (0.18 ± 0.08 vs. 0.42 ± 0.21) compared to ductal carcinomas in situ (DCIS) and invasive carcinomas with associated DCIS (*p* = 0.03). MDD enabled qualitative and quantitative evaluation of the composition of breast cancers and healthy glands.

## 1. Introduction

The continuous evolution and versatility of diffusion-weighted imaging (DWI) have made this technique a valuable tool that is increasingly used in breast cancer imaging with a wide variety of applications [1]. DWI non-invasively probes the random thermal motion of water molecules in vivo over the micrometer length scale and millisecond timescale. Such motion is more commonly referred to as “diffusion”. The resulting diffusion pattern of water molecules is shaped by the local tissue microstructure, e.g., different cellular densities, shapes, orientations, and membrane permeabilities. In pathological states such as cancer, these parameters are altered, which is reflected in the water mobility.

Current clinical conventional DWI methods, such as trace DWI, quantify water mobility in terms of a single apparent diffusion coefficient (ADC) per imaging voxel, which inversely reports on voxel-averaged cell density [2,3]. However, these methods fail to discriminate between the various mechanisms contributing to water mobility over the millimeter scale of typical DWI voxels [4]. Indeed, single ADCs per voxel cannot capture the non-Gaussian diffusion characterizing “heterogeneous tissues”, i.e., tissues comprising distinct cell types and orientations [5,6,7]. Another conventional approach to interpreting DWI is diffusion tensor imaging (DTI) [8]. This method describes tissues in terms of a single diffusion tensor averaged over the voxel scale. While the trace of this diffusion tensor, i.e., the mean diffusivity (MD), corresponds to the previously mentioned ADC, the anisotropy of this diffusion tensor corresponds to an additional biomarker: the fractional anisotropy (FA) [3]. This latter diffusion measure, reporting on the average anisotropy of the voxel content, confounds cell elongation and orientational order (degree of alignment of elongated cells) [9]. Consequently, FA vanishes in voxels containing complex crossing configurations of fibrous tissue, as their diffusion profiles appear isotropic once averaged over the voxel scale. The implications of this limitation, especially relevant in the central nervous system, are still unclear in the breast. Besides, DTI cannot capture tissue heterogeneity via its single voxel-averaged diffusion tensor [10]. Alternatively, diffusion kurtosis imaging (DKI) has enabled researchers to delve into tissue heterogeneity by capturing a normal distribution of diffusion coefficients [11,12,13]. In addition to providing the aforementioned MD, this approach employs higher b-values to estimate the mean kurtosis (MK), which reports on global microstructural tissue heterogeneity, i.e., a lack of internal organization [14]. Although MK has been shown to be superior to ADC or MD for lesion differentiation in the breast [15,16,17], it still confounds two sources of tissue heterogeneity, namely the variance of cell densities over the voxel scale and the presence of anisotropic cells, whether these are aligned or not over the voxel scale. Although other approaches have revealed nonparametric cell size distribution, this limitation has not been addressed yet [18,19].

The lack of specificity of the aforementioned conventional techniques used to interpret DWI data has recently been overcome within the framework of multidimensional diffusion (MDD) MRI [20,21,22]. This framework, inspired by the field of solid-state nuclear magnetic resonance (NMR) [23,24], relies on novel diffusion gradient waveforms to acquire complementary pieces of diffusion information that, once combined, enable the disentanglement of various tissue properties. Indeed, MDD measurements generalize the concept of diffusion encoding from the conventional b-value b and diffusion-sensitized orientation (Θ, Φ) to more versatile b-tensors b [22,25,26,27,28]. While the trace and main orientation of the b-tensor coincide with the b-value b and orientation (Θ, Φ), respectively, its anisotropy can be tailored to measure specific types of diffusion patterns. On the one hand, conventional diffusion MRI measurements correspond to linear b-tensors, only probing diffusion along their main orientation. On the other hand, non-trivial b-tensors consist of, e.g., planar b-tensors probing diffusion perpendicularly to their main orientation and spherical b-tensors probing isotropic diffusion. From a clinically relevant standpoint, MDD has so far been employed mostly in the brain, using more general truncated cumulant expansions of the diffusion signal [20,22,29]. The early results using microstructural information from MDD showed promising results in differentiating meningiomas and glioblastomas [30,31,32], differentiating cortex and white matter in malformations of cortical development associated with epilepsy [33], and characterizing white-matter lesions linked to multiple sclerosis [34] and Parkinson’s disease [35]. Diffusion tensor distribution (DTD) imaging [36] can be further applied to MDD data to estimate nonparametric intra-voxel distributions of diffusion tensors [37,38] via a quasi-genetic algorithm. DTD imaging has shown potential in the healthy brain to quantify various microstructural diffusion properties and differentiate tissue-specific subparts [39,40,41].

In this work, we hypothesized that acquiring MDD data and fitting the signal using DTD imaging allows for a more refined, non-invasive microstructural characterization of normal and neoplastic breast tissue through new quantitative imaging biomarkers. Therefore, the aim of this prospective pilot MDD was to investigate the potential of clinical multidimensional diffusion (MDD) MRI for the microstructural characterization of breast cancers and normal fibroglandular breast tissue (FGT).

## 2. Materials and Methods

### 2.1. Patients

This prospective Institutional Review Board-approved study was compliant with the Health Insurance Portability and Accountability Act (HIPAA), and was performed according to the principles of the Declaration of Helsinki. All participants gave their written informed consent for inclusion in the study.

From October 2019–March 2020, 16 consecutive women (mean age: 51.1 years, age range: 32–76 years) with newly diagnosed or clinically suspected breast cancer were included. All these women underwent a breast multiparametric MRI. The exclusion criteria of the study were: pregnancy or breastfeeding, contraindications to MRI or gadolinium-based contrast agents, and no previous breast surgery or treatment (i.e., neoadjuvant chemotherapy). There is no subject overlap with previously published studies in the literature.

### 2.2. MRI Examination Protocol

All MRI examinations were performed using a 3T magnet (Discovery MR750; GE Healthcare, Milwaukee, WI) with a dedicated 16-channel phased-array breast coil (Sentinelle Vanguard, Toronto, ON, Canada). The multiparametric MRI protocol comprised axial T1-weighted non-fat saturated, axial T2-weighted fat saturated images, and T1-weighted fat saturated axial images before and after contrast injection (0.1 mmol Gadavist/kg body weight). In addition, axial DW (diffusion-weighted) images were acquired after contrast injection. First, conventional DW echo planar imaging (EPI) was acquired with two b-values (0, 800 s/mm^2^) and 3 encoding directions. Subsequently, MDD was acquired using a custom DW sequence (spin echo-prepared EPI sequence using TE = 98 ms, TR = 2.7–5.0 s, FOV = 35 × 35 cm, matrix size = 128 × 128, in-plane resolution = 2.7 mm, slice thickness = 5 mm, and scan time ≈ 4 min). Five sets of b-values (0, 100, 700, 1400, and 2000 s/mm^2^) were acquired using gradient waveforms targeting 37 isotropic linear encoding directions and 43 spherically encoded signals. A maximum of 35 slices were individually adjusted to cover the whole breast. Technical details of the MRI protocol are shown in Table 1.

### 2.3. Image Assessment and Data Collection

Images were reviewed by a breast dedicated radiologist with 5 years of experience (I.D.N.) using Centricity^TM^ universal viewer (GE Healthcare, IL, USA). Cancers were identified on dynamic contrast-enhanced (DCE) images and the slice location was recorded to match DW images. The largest lesion axis measurements and the amount of FGT were determined by DCE series according to the American College of Radiology Breast and Imaging Reporting and Data System (BI-RADS) [42]. Clinical history was reviewed to determine age and menopausal status. Histopathology results were reviewed for cancer histology, histological grade, and immunohistochemistry (IHC) status [43]. Evaluation of IHC status included estrogen (ER), progesterone (PR), and human epidermal growth factor receptor 2 (HER2) status according to the standard protocols using an automated Ventana Benchmark XT device (Ventana, Tucson, AZ, USA). The reference standard was histological analysis of the surgical specimen; in patients who received neoadjuvant treatment, the results from image-guided biopsies were considered the reference standard.

### 2.4. Diffusion Tensor Distributions (DTDs) and DTD-Derived Maps

**Figure 1:**
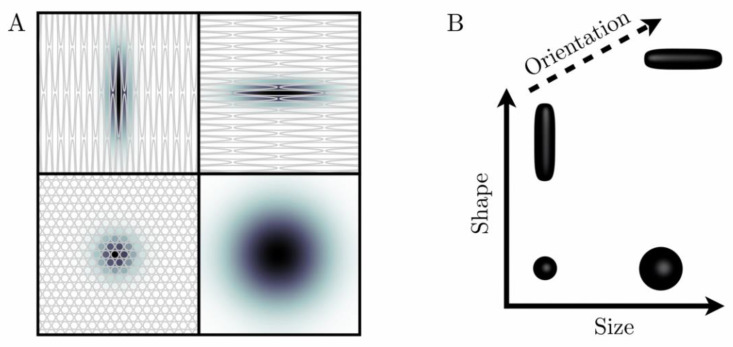
“Ink drop” analogy to describe and parametrize microscopic diffusion patterns (**A**). When ink drops into a medium, it diffuses over time and leaves a stain (diffusion pattern), the size and shape of which are conditioned by the microstructure of the medium. Grey objects correspond to rather permeable cell membranes (**B**). Diffusion patterns are mathematically described by diffusion tensors **D**, which can be represented geometrically by glyphs (black three-dimensional objects) shaped like the corresponding diffusion patterns. In particular, the size, shape, and orientation of these glyphs are given by the trace of **D**, the variance of the eigenvalues of **D**, and the main eigenvector of **D**, respectively.

**Figure 2:**
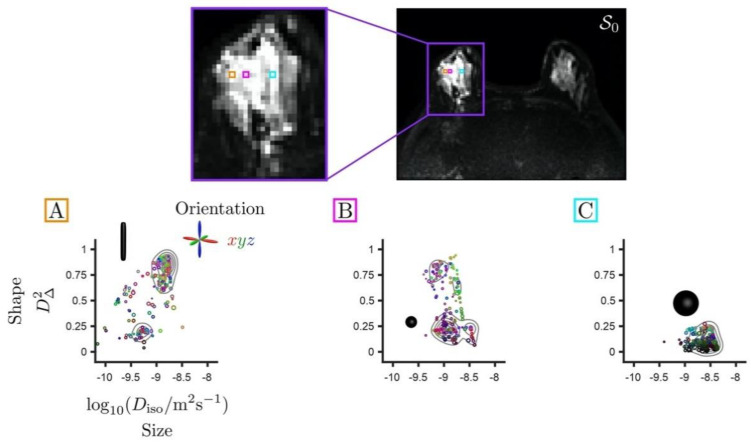
Set of diffusion tensor distributions (DTDs) for individual voxels extracted from a T2-weighted non diffusion-weighted *S*_0_ map with an invasive ductal carcinoma in the right breast. The distributions are represented in two-dimensional plots of diffusion tensor sizes (*D*_iso_) and shapes (*D*_∆_^2^). Size (*D*_iso_) reports on cellularity in an inversely proportional relation. Shape (*D*_∆_^2^) values range from 0 for spherical tensors to 1 for elongated tensors. Voxels (**A**,**B**) including cancer exhibit diffusion in densely packed elongated cells and tumoral isotropic cells. Voxel (**C**) presents isotropic cells with higher diffusivity corresponding to healthy fibroglandular tissue. The directional color-coding is based on the diffusion tensor eigenvalues, normalized by the maximum eigenvalue, and reports on the orientation of the underlying diffusion patterns. The red/green/blue directions correspond to the left-right/anterior-posterior/superior-inferior directions, respectively. The key diffusion properties of these cellular configurations can be quantified via the statistical descriptors of the DTD, i.e., means and (co)variances calculated over the size (*D*_iso_), shape (*D*_∆_^2^), and orientation dimensions of the DTD. The diffusion encoding strategies offered by multidimensional diffusion (MDD) acquisitions enable simultaneous measurement of various features of the DTD, thereby enhancing the specificity of the microstructural information arising from diffusion processes in vivo.

The biological content of a given imaging voxel is commonly described by a diffusion tensor distribution (DTD), wherein each diffusion tensor **D** encapsulates the “size”, “shape”, and “orientation” of a microscopic diffusion pattern [37,38]. The DTD also corresponds to the joint distribution of quantities that specifically reflect these diffusion dimensions. In particular, for a given diffusion tensor with axial diffusivity $D_{∥}$ and radial diffusivity $D_{⊥}$, the isotropic diffusivity $D_{\mathrm{iso}}=\left(D_{∥}+2D_{⊥}\right)/3$ and the diffusion anisotropy $D_{\Delta }^{2}={\left[\left(D_{∥}-D_{⊥}\right)/\left(3D_{\mathrm{iso}}\right)\right]}^{2}$ [44] are associated with the “size” and “shape” dimensions, respectively. Importantly, the “size” of a diffusion tensor is proportional to the inverse cell density in a given tissue, and is not related to cell size per se. While Figure 1 illustrates the correspondence between local tissue geometry and diffusion tensor properties, Figure 2 presents DTDs in the context of breast healthy FGT and cancers.

MDD gradient waveforms allow for the simultaneous measurement of various features of these voxel-wise DTDs in a unique way [21,27]. In particular, DTD imaging [36] employs MDD data to estimate nonparametric intra-voxel DTDs. These distributions are obtained via a quasi-genetic approach detailed in previous works [39,40,45]. Let us briefly describe this approach. A set of 200 diffusion tensors (components) is randomly generated and its components’ likelihood to explain the measured signals is assessed via non-negative least-squares (NNLS) fitting [46]. After repeating this so-called “proliferation” step 20 times, the remaining components with non-zero weight are randomly perturbed during the “mutation” step. Original and perturbed components are then competing with one another on the NNLS basis, with largest-weight solutions kept at the end of this “extinction” step. After repeating the mutation and extinction steps 20 times, the 50 largest-weight components form the final solution (or DTD). The entire procedure is repeated 100 times using bootstrap with replacement [47,48] on the measured signal. Statistical descriptors of the DTD, e.g., means E[ · ] and variances V[ · ] calculated over the tensor size (*D*_iso_), shape (*D*_∆_^2^), and orientation dimensions of the DTD can then be computed as medians across these bootstrap solutions [45].

The mean size E[*D*_iso_] corresponds to the conventional mean diffusivity and is proportional to the inverse cell density. The mean shape E[*D*_∆_^2^] captures microscopic anisotropy, i.e., the mere presence of elongated cells, whose values range from 0 for spherical tensors to 1 for completely elongated tensors. The variance of diffusion tensor size V[*D*_iso_] quantifies the variance of cell densities over the voxel scale. The 10th percentile values of the mean diffusion tensor size, denoted by (E[*D*_iso_])_10%_, can be calculated within tumors to capture the most diffusion-restrictive areas. DTD imaging also retrieves the conventional FA and the orientational order parameter (OP), defined as the ratio (FA/µFA)^2^ between the FA and the microscopic fractional anisotropy µFA [20,22]. OP reflects the degree to which elongated cells align together over the voxel scale, ranging from 0 for randomly oriented cells to 1 for perfectly aligned cells. In other words, DTDs allow for the quantification of the composition and orientational order of heterogeneous tissues, such as the breast healthy FGT and cancerous lesions of Figure 2.

**Figure 3:**
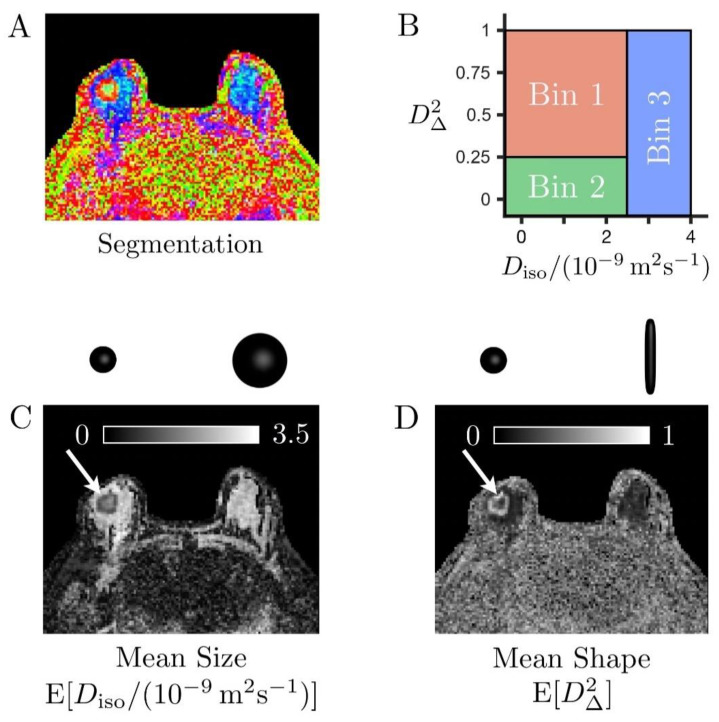
Binning of the size–shape space of the diffusion tensor distributions (DTDs) in a case of invasive ductal carcinoma (**A**). Color-coded map derived from bin-resolved signal fractions, respectively associated with elongated cells (bin1, red), isotropic diffusion environments with low diffusivity (bin2, green), and isotropic diffusion environments with high diffusivity (bin3, blue) (**B**). Definition of the tissue-specific bins in the two-dimensional plane of tensor size *D*_iso_ and squared tensor shape *D*_∆_^2^ (**C**,**D**). Grey scale maps reporting on the mean size E[*D*_iso_] and mean shape E[*D*_∆_^2^] of the entire voxel content, respectively. The cancer (white arrows) exhibits high cellularity (low mean size E[*D*_iso_]) compared to the healthy fibroglandular tissue (large blue and cyan areas indicated by the yellow arrow in (**A**). It also features prominent heterogeneity, with a core consisting of slow-diffusive isotropic environments (large *f*_bin2_, low mean shape E[*D*_∆_^2^]), which could correspond to densely packed isotropic cells or necrotic tissue, surrounded by a layer of elongated cells (large *f*_bin1_, high mean shape E[*D*_∆_^2^]).

Moreover, the size–shape space of these distributions can be binned to isolate signal fractions from elongated cells (*f*_bin1_), isotropic diffusion environments with low diffusivity (*f*_bin2_), and large isotropic diffusion environments with high diffusivity (*f*_bin3_) [27,39,41]. The result of this binning procedure is illustrated in Figure 3 using a color-coded segmentation map representing the different proportions of these various tissue types within each voxel. The bins were defined as follows:

- Bin 1 within $D_{\mathrm{iso}}\in \left[0,\;2.5\right]\;{\mu m}^{2}/\mathrm{ms}$ and $D_{∥}/D_{⊥}\in \left[4,1000\right],$
- Bin 2 within $D_{\mathrm{iso}}\in \left[0,\;2.5\right]\;{\mu m}^{2}/\mathrm{ms}$ and $D_{∥}/D_{⊥}\in \left[0.01,\;4\right]$,
- Bin 3 within $D_{\mathrm{iso}}\in \left[2.5,\;10\right]\;{\mu m}^{2}/\mathrm{ms}$ and $D_{∥}/D_{⊥}\in \left[0.01,\;1000\right].$

Bin-specific statistical descriptors were estimated following the above process for the retrieved components specifically falling into each bin. Note that this manual binning corresponds to the one typically performed in the healthy brain [27,39,41], and was directly translated to the breast in this work, with no data-driven optimization. In particular, the $D_{\mathrm{iso}}=2.5\;{\mu m}^{2}/\mathrm{ms}$ bound typically yields a good separation between cerebrospinal fluid and white/grey matter, and the $D_{∥}/D_{⊥}=4$ bound corresponds to the diffusion anisotropy $D_{\Delta }=0.5$ and the fractional anisotropy FA=0.7. In other words, this binning procedure consists merely of a preliminary attempt to comprehend the rich information contained in the DTD and is, as such, a limitation that could be mitigated by automatic clustering methods or by higher dimensional versions of data-driven techniques, such as those which have been previously published [49,50].

The raw MDD images were corrected for motion and eddy currents using the extrapolation-based references method [51]. Further processing in a per-voxel manner using the DTD imaging algorithm [36] was implemented in dVIEWR powered by MICE Toolkit^TM^ (Random Walk Imaging AB and NONPI Medical AB, Sweden, www.dviewr.com and www.micetoolkit.com) (accessed on 29 March 2021) to quantify intravoxel tissue composition in terms of the aforementioned DTD statistical descriptors. 

### 2.5. Quantitative Analysis of the Maps

According to the location and lesion dimensions on DCE images, cancers were identified in dVIEWR on *b* = 700 s/mm^2^ images and multiparametric maps, and a volumetric region of interest (ROI) was manually drawn. ROIs containing more than 10 voxels were placed in locations classified as FGT in the healthy breast, and as cancer on areas with correlative tumor confirmed by DCE images. In the case of lesions with enhancing and non-enhancing components, a whole-tumor ROI was drawn with the intention of capturing distinguishable features between the various estimated DTD statistical descriptors.

### 2.6. Statistical Analysis

Statistics for nominal data were reported using percentages and absolute values, and metrics data were reported using mean ± standard deviation. Statistical software SPSS 24.0 (IBM Corp., Chicago, IL, USA) was used to perform the statistical analysis. The Mann–Whitney U test was used to analyze differences in metrics between the study participants and biological tissues. *p* < 0.05 was considered indicative of a significant difference. 

## 3. Results

### 3.1. Patient Cohort and Lesion Characteristics

Sixteen women with 16 breast cancers (mean size 30 mm, range 6–80 mm) were analyzed. A review of the images and clinical history revealed eight post-menopausal and eight pre-menopausal women with predominantly dense breast (almost entirely fatty breast = 1, scattered FGT = 3, heterogeneous FGT = 8 and extreme amount of FGT = 4). There were two ductal carcinomas in situ (DCIS), one invasive lobular carcinoma (ILC), eight invasive ductal carcinomas (IDC), and five IDCs with associated extensive DCIS component. Among these cancers, eight were grade 3, seven were grade 2 and one was grade 1. Among invasive carcinomas, 12 were positive for ER, 11 were positive for PR, and 4 were positive for HER2. Clinicopathologic characteristics of the patients and lesions are shown in Table 2.

### 3.2. Diffusion Tensor Distributions (DTDs) Results

**Figure 4:**
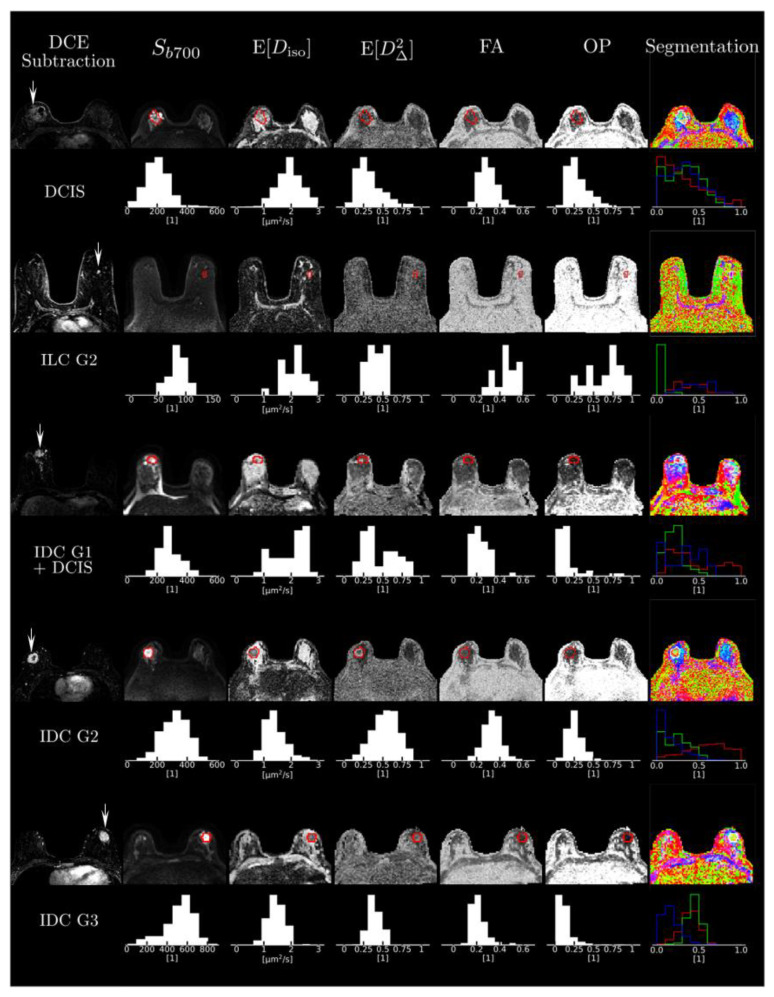
Cases of four representative cancers. Figure shows subtracted dynamic contrast-enhanced images and the different multidimensional diffusion (MDD)-derived parametric maps for each case with corresponding histograms. Abbreviations: DCE, dynamic contrast-enhanced; DCIS, ductal carcinoma in situ; FA, fractional anisotropy; G, grade; IDC, invasive ductal carcinoma; OP, orientational order parameter.

The mean size of the voxels analyzed was 4.23 ± 4.21 cubic centimeters for cancers and 2.97 ± 1.24 cubic centimeters for FGT (*p* = 0.92). The visual analysis of the parametric maps showed that tumors were consistently characterized by high signal intensity *S_b_*_700_ in the *b* = 700 s/mm^2^ images, i.e., hindered diffusivity associated with a low mean size E[*D*_iso_], and a high mean shape E[*D*_∆_^2^] corresponding to bin1, as shown on Figure 4. Importantly, all cancer types were invisible in the conventional FA maps. This is due to the fact that they featured a low orientational order parameter OP [20], reflecting the random orientation of their elongated-cell components over the voxel scale.

**Figure 5:**
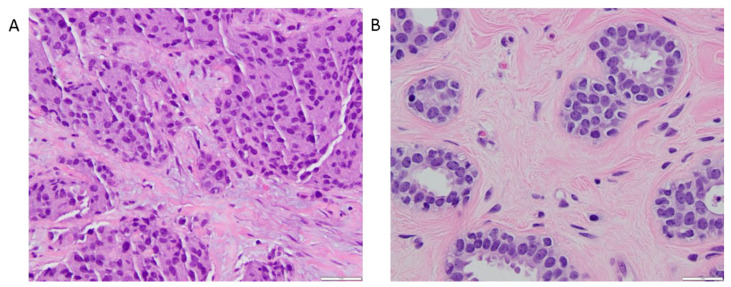
400× magnified hematoxylin and eosin-stained sections. (**A**) 39 mm microscopic invasive ductal carcinoma corresponding to the one shown in Figure 3. The anatomopathological section shows moderately pleomorphic cells with a majority exhibiting irregular and oval shape. (**B**) Benign breast parenchyma adjacent to the tumor in Figure 5A. In contrast to the large, irregular nuclei in the tumor cells, the nuclei in the cells forming the benign mammary acini are round and uniformly smooth. Mean diffusion tensor size (E[*D*_iso_]) value in this cancer was 1.09 × 10^−3^ mm^2^/s, compared to 2.32 × 10^−3^ mm^2^/s for the healthy glandular tissue, reflecting a higher cell density within the neoplastic section. Mean diffusion tensor shape (E[*D*_∆_^2^]) value in this carcinoma was 0.64, compared to 0.26 for the healthy glandular tissue, which denotes predominantly elongated components within the cancerous tissue section.

The various DTD-derived metrics obtained from tumor ROI analysis and within FGT are summarized in Table 3. The whole tumor ROI histogram analysis of the DTD-derived maps for the tumors exhibited the mean diffusion tensor shape E[*D*_∆_^2^] = 0.47 ± 0.15 and the mean diffusion tensor size E[*D*_iso_] = (1.43 ± 0.54) × 10^−3^ mm^2^/s, corresponding to a preponderance of elongated cells, as captured by the signal fraction of bin1, *f*_bin1_ = 0.53 ± 0.27. Figure 5 shows an example of a hematoxylin and eosin-stained section of an invasive ductal carcinoma with a predominance of irregular and oval-shaped cells, and healthy fibroglandular tissue with a predominance of round and uniformly smooth cells.

The variance of isotropic diffusivities in the tumors was V[*D*_iso_] = (0.73 ± 0.19) × 10^−6^ mm^4^/s^2^ (maps not shown in the figures). On the contrary, the healthy FGT yielded higher values of E[*D*_iso_] and lower values of E[*D*_∆_^2^], which corresponds to a preponderance of fast-diffusing, i.e., non-hindered isotropic environments, as captured by the signal fraction of bin 3, *f*_bin3_ = 0.62 ± 0.1. The variance of isotropic diffusivities in FGT was V[*D*_iso_] = (0.97 ± 0.33) × 10^−6^ mm^4^/s^2^.

Cancers and normal FGT were significantly different according to all the metrics except for the variance of isotropic diffusivities V[*D*_iso_], OP and *f*_bin2_, which is associated with densely packed isotropic cells or slow-diffusing isotropic environments. The color-coded segmentation map derived from bin-resolved signal fractions in Figure 3 indeed shows that both cancers and FGT can contain this tissue type.

We previously reported values for the 10th percentile of mean diffusion tensor size within tumors, denoted by (E[*D*_iso_])_10%_ [52]. The mean value for this parameter was (1 ± 0.46) × 10^−3^ mm^2^/s. When comparing purely invasive carcinomas with mixed invasive and in situ carcinomas and DCIS tumors in this study, the differences in the mean values of (E[*D*_iso_])_10%_ were not significant: (0.88 ± 0.28) × 10^−3^ mm^2^/s versus (1.17 ± 0.62) × 10^−3^ mm^2^/s, respectively (*p* = 0.11). However, signal fractions from bin1 and bin3 did show significant differences: *f*_bin1_ = 0.64 ± 0.13 for invasive tumors and *f*_bin1_ = 0.4 ± 0.25 for the second group (*p* = 0.03), and *f*_bin3_ = 0.18 ± 0.08 for invasive tumors and *f*_bin3_ = 0.42 ± 0.21 (*p* = 0.03) for the second group. DTD-derived metrics and comparisons based on tumor histopathology are reported in Table 4.

The metrics obtained from the histogram analysis for healthy FGT were compared based on the menopausal status of the participants. No significant differences were found with any of the metrics. DTD-derived metrics and comparisons based on menopausal status are reported in Table 5.

The small number of observations within categories for tumor grade, HER2, and hormonal receptor status prevented more detailed correlation of DTD-specific metrics with these features of invasive cancers.

## 4. Discussion

In this prospective pilot study, we investigated the potential of clinical MDD MRI for the microstructural characterization of breast cancers and normal fibroglandular breast tissue. Our results showed that cancers were characterized by low E[*D*_iso_] and high E[*D*_∆_^2^], corresponding to bin1 (elongated cells), while normal FGT exhibited high E[*D*_iso_] and low E[*D*_∆_^2^], corresponding to bin3 (fast-diffusing, i.e., non-hindered isotropic environments). When comparing pure invasive carcinomas with mixed invasive and ductal in situ carcinomas and DCIS tumors, signal fractions from bin1 and bin3 were significantly different, with *f*_bin1_ typically higher than *f*_bin3_, suggesting the predominance of elongated cells in invasive carcinomas. Maps allowed qualitative and quantitative evaluation of the composition and orientational order of healthy breast FGT and heterogeneous cancerous tumors.

Conventional imaging biomarkers, MD and ADC, that are commonly used in clinical practice to differentiate healthy tissue, benign lesions, and malignant lesions [52] are equivalent to the DTD-derived mean size E[*D*_iso_]. Previous observations in breast lesions have reported that MD is inversely correlated with tissue cellularity. The observations of MD in breast tumors and FGT in previous publications [53,54] are in agreement with our results, wherein the E[*D*_iso_] values in cancers were significantly lower than in FGT due to higher cellularity. Our values for E[*D*_iso_] in FGT are in line with previous MD observations, with values over 2 × 10^−3^ mm^2^/s [55,56,57]. Furthermore, our E[*D*_iso_] values in cancers are within the bracket of those reported for MD in malignant tumors in a recent meta-analysis by Wang et al. [58]. Although MD values have been shown to be reproducible, it is worth noting that Wang et al. reported a wide range of MD values in tumors across different studies, ranging from 0.71 × 10^−3^ mm^2^/s to 1.62 × 10^−3^ mm^2^/s, which may be explained by different histopathologies and growth patterns that are not evaluable with conventional DWI [58,59]. Therefore, new DTD-derived mean size E[*D*_iso_] may provide more consistent and reproducible results, as DTD imaging [36] accounts for the non-Gaussian diffusion effects originating from the existence of multiple cell densities, shapes, and orientations below the imaging voxel scale in biological tissues.

Both MD and ADC, which correspond to the same biomarker of inverse cell density, have been used interchangeably in the literature [54,60,61], and are employed in clinical practice to distinguish between benign and malignant breast lesions. While malignant lesions are typically identified as regions of low ADC, it is important to note that diagnostic accuracy is highly dependent on the choice of an appropriate threshold below which ADC is considered “low”. While the European Society of Breast Imaging (EUSOBI) International Breast Diffusion-Weighted Imaging working group advocates for a conservative ADC threshold of 1.3 × 10^−3^ mm^2^/s to indicate lesion suspiciousness, a recent meta-analysis based on 13,847 lesions by Surov et al. recommends a cut-off value of 1 × 10^−3^ mm^2^/s [52,62]. What seems to be clearer is the fact that minimum ADC values with small ROIs yield the best results [52]. Bickel et al. reported that minimum ADC values and those obtained from 2D ROIs showed the best diagnostic performance [63]. Similarly, Arponen et al. reported that the use of small ROIs achieved better accuracies than the use of a whole-tumor approach. In addition, they reported that cut-off values differed significantly depending on the measurement procedure [64]. Following this evidence, and according to recommendations from the EUSOBI International Breast Diffusion-Weighted Imaging working group, we reported values for the 10th percentile (E[*D*_iso_])_10%_ of the DTD-derived mean size within tumors to reflect the most active part of the tumors without being overly sensitive to outliers [63]. In this study, no significant differences were found with this parameter between invasive carcinomas and the group of mixed carcinomas and DCIS tumors. These results denote that the approach used in our study surely captured the most aggressive components of the tumors, since most of the cancers in the second group contained infiltrative components to some extent. This may have an impact on treatment planning and biopsy guidance. Nevertheless, it is worth mentioning that, given the significance of values obtained, a comparison with a bigger sample of DCIS would be desirable to fully evaluate the potential of this biomarker to differentiate invasive from non-invasive cancers.

FA values confound cell elongation and cell orientational order (alignment) by capturing the average anisotropy of the voxel content [9]. While the DTD-derived mean shape E[*D*_∆_^2^] also reports on tissue anisotropy, it does so without being influenced by the orientational order of fibrous tissues at the sub-voxel scale (quantified by the orientational order parameter OP). We found significantly higher mean values of FA and E[*D*_∆_^2^] in tumors compared to FGT, agreeing with the previous literature on FA [55,65], which indicates that both metrics could be appropriate for breast cancer diagnosis. However, the mean shape E[*D*_∆_^2^] appears to be a more reliable metric than FA, as shown by its higher values in tumors with low OP and its higher statistical power for distinguishing tumors and FGT (*p* < 0.001 for E[*D*_∆_^2^] versus *p* = 0.02 for FA).

Low E[*D*_∆_^2^] and FA values throughout healthy breast tissue may indicate that the abundance of elongated structures such as ducts, lobules, and stroma in FGT have larger diameters than the mean displacement of water molecules during the diffusion time [55] probed by our acquisition gradient waveforms. While theoretical tools are currently being developed to further investigate diffusion time-dependent effects [18,19] in the context of MDD acquisitions [27,66], such investigations go beyond the scope of the present work.

Several studies have reported lower diffusion coefficients and higher anisotropy indices in elderly volunteers, probably secondary to a decrease in water content and an increase in adipose tissue [55,67,68]. Like observations reported by Cakir et al. [54], our analysis did not show significant differences in metrics between premenopausal and postmenopausal participants in the healthy breast. This could be due to the age range of the participants around the perimenopausal period.

From a heterogeneity standpoint, our study showed that MDD alleviates the limitations of DKI in the breast, teasing apart the two microstructural sources of mean kurtosis: the variance of isotropic diffusivities and the microscopic anisotropy. Indeed, we found significant differences in mean diffusion tensor shape (E[*D*_∆_^2^]) between tumors and FGT, whereas no differences were found in the variance of isotropic diffusivities V[*D*_iso_].

This current study has some limitations. Firstly, the in-plane resolution of this sequence is low in comparison with other DWI encoding methods. This can influence the delineation of lesions, especially small ones. A combination of these advanced MDD gradients with high resolution DWI encoding strategies would be desirable. Secondly, the MDD sequence was performed after contrast injection, which might produce a bias towards tissues which enhance after contrast (i.e., tumors), reducing measured ADC values; however, this has been shown to have no significant impact on overall breast cancer diagnostic performance [69,70]. Thirdly, we acknowledge that the small sample size limited the conclusion on tumor grading or molecular profiles of the tumors; yet, this was a prospective pilot study designed to demonstrate clinical feasibility. Fourthly, although our choice of DTD bins yielded consistent results among the cases investigated in this work, their manual setting may limit generalizations to larger patient cohorts. Instead, efforts should be made towards developing data-driven ways of identifying these bins. Promising approaches consist of automatic clustering methods, such as the density peak clustering method [71], which was recently combined with DTD [72], and higher dimensional versions of previously published data-driven techniques [49,50]. Note, however, that the mean shape E[*D*_∆_^2^], identified in this work as a potential biomarker of microscopic anisotropy, remains unaffected by binning. Lastly, although the validity of MDD in brain tumors has been confirmed in recent correlative studies with histopathology [31], we acknowledge that our initial results of this pilot study in breast tumors will require further validation in preclinical and clinical studies in which whole tumor histological specimens are carefully co-registered to in vivo imaging.

## 5. Conclusions

Our prospective pilot study for MDD MRI imaging in breast cancer patients indicates the potential for the more specific characterization of breast cancer through additional quantitative imaging biomarkers with intuitive relations to the underlying tissue and cell structure. These new metrics, computed using DTD imaging, quantify fundamentally different microstructural properties that are inextricably entangled, and thus not available for cancer characterization with conventional diffusion MRI. Although MDD requires customized sequences that rely on advanced gradient waveforms with both spherical and multidirectional linear encodings of the MR signal, the acquisition time is similar to conventional diffusion MRI, thus being suitable for easy clinical translation. The promising results in our prospective pilot study encourage further studies with larger patient groups and correlation with histopathology and immunohistochemistry.

## Figures and Tables

**Table 1:** Full breast axial 3T protocol, 16-channel breast array.

Sequences	1	2	3	4	5
Series descriptor	3D axial T2	3D axial T1	3D axial DISCO	2D axial DWI	2D axial MDD DWI
Sequence type	Fast-Spin Echo	Gradient-Echo VIBRANT	Gradient-Echo	Spin-Echo EPI	Spin-Echo EPI
Field of view (cm)	34–38	34–38	34–38	34–38	34–38
Slice thickness (mm)	3	1.1	1.1	3.9	3.9
Gap (mm)	3	0	0	0	2.7
Fat saturation	Yes	No	Yes	Yes	Yes
TE (ms)	Minimum	Minimum	Minimum	Minimum	98
TR (ms)	2500–6000	4–4.5	4–4.5	2000–6000	2700–5000
Matrix size (mm)	512 × 512	512 × 512	512 × 512	256 × 256	128 × 128
Flip angle	111	10	12	90	90
Direction				ALL	ALL
Number of directions				3	43
b-values (s/mm^2^)				0, 800	100, 700, 1400, 2000
Frequency direction	A/P	A/P	A/P	A/P	A/P
Scan time (min:sec)	2:32	1:30	5–6 ^1^	4:02	4:12

Abbreviations: A/P, anterior/posterior; DISCO, differential subsampling with Cartesian ordering; EPI, echo planar imaging; MDD, multidimensional diffusion; VIBRANT, volume imaging for breast assessment. ^1^ For a total of 10–13 phases with a temporal resolution of 12 ms.

**Table 2:** Clinicopathologic characteristics of patients and lesions.

Feature	Value
Patients (*n* = 16)	
Mean patient age in years (SD)	51.1 (13.5)
Menopausal status	
Pre-menopausal	8 (50%)
Post-menopausal	8 (50%)
Breast type	
Almost entirely fatty	1 (6.25%)
Scattered FGT	3 (18.25%)
Heterogeneous FGT	8 (50%)
Extreme FGT	4 (25%)
Breast tumors (*n* = 16)	
Size in mm (SD)	30 (17.5)
Lesion type on DCE-MRI	
Mass	12 (75%)
NME	2 (12.5%)
Mixed	2 (12.5%)
Histopathology	
IDC	8 (50%)
ILC	1 (6.25%)
DCIS	2 (12.5%)
IDCs with extensive DCIS component	5 (31.25%)

Abbreviations: DCE-MRI, dynamic contrast-enhanced magnetic resonance imaging; DCIS, ductal carcinoma in situ; FGT, fibroglandular tissue; IDC, invasive ductal carcinoma; ILC, invasive lobular carcinoma; *n*, number; NME, non-mass-enhancing lesion; SD, standard deviation.

**Table 3:** Mean values of DTD-derived metrics for tumors and FGT.

Metrics	Tumors	FGT	*p*-Value
Mean diffusion tensor size (E[*D*_iso_])	1.43 (0.54)	2.33 (0.22)	<0.001
Variance of diffusion tensor sizes (V[*D*_iso_])	0.73 (0.19)	0.97 (0.33)	0.06
Mean diffusion tensor shape (E[*D*_∆_^2^])	0.47 (0.15)	0.27 (0.11)	<0.001
Fractional anisotropy (FA)	0.39 (0.07)	0.32 (0.08)	0.02
Orientational order parameter (OP)	0.38 (0.16)	0.38 (0.17)	0.71
Signal fraction of bin 1 (*f*_bin1_)	0.53 (0.27)	0.17 (0.14)	<0.001
Signal fraction of bin 2 (*f*_bin2_)	0.23 (0.11)	0.22 (0.07)	0.40
Signal fraction of bin 3 (*f*_bin3_)	0.29 (0.19)	0.62 (0.10)	<0.001

While E[*D*_iso_] is expressed in 10^−3^ mm^2^/s, V[*D*_iso_] is expressed in 10^−6^ mm^4^/s^2^. Other coefficients are unitless. Numbers in brackets are standard deviations. FGT stands for fibroglandular tissue.

**Table 4:** Mean values of DTD-derived metrics for tumor histopathology.

Metrics	Invasive Tumors	DCIS and IDCs with Extensive DCIS Component	*p*-Value
Mean diffusion tensor size (E[*D*_iso_])	1.22 (0.32)	1.72 (0.66)	0.05
Variance of diffusion tensor sizes (V[*D*_iso_])	0.68 (0.16)	0.79 (0.22)	0.24
Mean diffusion tensor shape (E[*D*_∆_^2^])	0.53 (0.10)	0.4 (0.18)	0.11
Fractional anisotropy (FA)	0.38 (0.07)	0.4 (0.08)	0.45
Orientational order parameter (OP)	0.35 (0.15)	0.43 (0.17)	0.20
Signal fraction of bin 1 (*f*_bin1_)	0.64 (0.13)	0.4 (0.25)	0.03
Signal fraction of bin 2 (*f*_bin2_)	0.24 (0.12)	0.21 (0.11)	0.15
Signal fraction of bin 3 (*f*_bin3_)	0.18 (0.08)	0.42 (0.21)	0.03

While E[*D*_iso_] is expressed in 10^−3^ mm^2^/s, V[*D*_iso_] is expressed in 10^−6^ mm^4^/s^2^. Other coefficients are unitless. Numbers in brackets are standard deviations. Abbreviations: DCIS, ductal carcinoma in situ; IDC, invasive ductal carcinoma.

**Table 5:** Mean values of DTD-derived metrics for menopausal status.

Metrics	Premenopausal	Postmenopausal	*p*-Value
Mean diffusion tensor size (E[*D*_iso_])	2.35 (0.22)	2.31 (0.23)	0.96
Variance of diffusion tensor sizes (V[*D*_iso_])	0.88 (0.3)	1.06 (0.36)	0.42
Mean diffusion tensor shape (E[*D*_∆_^2^])	0.25 (0.09)	0.29 (0.13)	0.71
Fractional anisotropy (FA)	0.3 (0.07)	0.33 (0.09)	0.37
Orientational order parameter (OP)	0.36 (0.18)	0.4 (0.18)	0.42
Signal fraction of bin 1 (*f*_bin1_)	0.14 (0.11)	0.20 (0.17)	0.42
Signal fraction of bin 2 (*f*_bin2_)	0.23 (0.08)	0.21 (0.06)	0.96
Signal fraction of bin 3 (*f*_bin3_)	0.63 (0.11)	0.61 (0.10)	0.87

While E[*D*_iso_] is expressed in 10^−3^ mm^2^/s, V[*D*_iso_] is expressed in 10^−6^ mm^4^/s^2^. Other coefficients are unitless. Numbers in brackets are standard deviations.

## Data Availability

The data presented in this study are available upon reasonable request from the corresponding author.
